# Efficacy of a Stannous-containing Dentifrice for Protecting Against Combined Erosive and Abrasive Tooth Wear In Situ

**DOI:** 10.3290/j.ohpd.a44926

**Published:** 2020-07-24

**Authors:** Xinyi Zhao, Tao He, Yanyan He, Haijing Chen

**Affiliations:** a Professor, School of Stomatology, the Fourth Military Medical University, Shanxi, China. Study design, executed the study, interpreted the data, co-wrote the manuscript, approved the manuscript for submission.; b Principal Clinical Scientist, The Procter & Gamble Company, Mason, OH, USA. Study design, interpreted the data, drafted the manuscript, approved the manuscript for submission.; c Statistician, Procter & Gamble Technology Beijing Company Ltd, Beijing, China. Study design, statistical analysis, interpreted the data, co-wrote the manuscript, approved the manuscript for submission.; d Senior Scientist, Procter & Gamble Technology Beijing Company Ltd, Beijing, China. Executed the study, interpreted the data, co-wrote the manuscript, approved the manuscript for submission.

**Keywords:** dental erosion, dentifrice, erosive tooth wear, in situ clinical, stannous fluoride

## Abstract

**Purpose:**

The in-situ efficacy of an experimental stannous (Sn)-containing sodium fluoride (NaF) dentifrice against erosion and erosive tooth wear was compared with a conventional NaF dentifrice.

**Materials and Methods:**

This was a randomised, controlled, double-blind, parallel-group clinical trial. Mandibular appliances containing four enamel specimens (2 per side [L/R] of the appliance) were worn by 60 generally healthy adult subjects. Subjects were randomised to treatment based on age and gender. Treatments included a Sn-containing NaF or conventional NaF dentifrice. Conditions of erosion (dentifrice slurry treatment) and erosion/tooth wear (dentifrice slurry plus brushing) were compared. Dentifrices were used twice per day for 30 s of lingual brushing, followed by 90 s of slurry exposure. In addition, the two specimens on the left side of the mouth were brushed for 5 s each, using a power toothbrush. All specimens were exposed to four daily erosive challenges with commercial orange juice (pH 3.6). Tooth wear was measured as enamel loss using non-contact profilometry on day 10.

**Results:**

At the day 10 visit, the adjusted mean (SE) enamel loss for specimens receiving slurry (erosion) treatment was 4.7 µm (0.61) [Sn-containing NaF] and 8.73 µm (1.12) [NaF control], with results demonstrating a statistically significant benefit for the Sn-containing dentifrice (46.2% benefit; p = 0.009). For specimens exposed to erosion/tooth wear conditions, enamel loss = 6.68 µm (1.29) (Sn-containing NaF) and 10.99 µm (1.29) (NaF group), with results statistically significant (p = 0.048; 39.2% better, favouring the Sn-containing dentifrice). When data were combined, enamel loss (SE) for all specimens subjected to erosion + erosion/tooth wear was 5.61 µm (0.77) (Sn-containing NaF]) and 9.9 µm (1.3) (NaF group). The difference again was statistically significant, favouring the Sn-containing group (p = 0.022; 43.4% better).

**Conclusions:**

The Sn-containing dentifrice demonstrated significantly better protection than did NaF under erosive and erosive/tooth wear conditions.

Dental erosion has received widespread attention over the last several years, largely because of the general irreversibility of the condition.^28^ Although dental erosion is commonly associated with the excessive consumption of acidic foods and beverages, which has dramatically risen in recent times,^44^ other factors, such as lifestyle and medical conditions have also been shown to increase the potential for dental erosion.^12,26,34^ The impact of these dietary and lifestyle changes on the prevalence of erosion is reflected in more recent publications. For example, a recent meta-analysis based on the results of 22 studies found a prevalence of erosive wear in the permanent teeth of children and adolescents to be 30.4%.^35^

Under ideal conditions, permanent teeth should last a lifetime. However, oral diseases, such as caries and periodontal disease, significantly reduce the clinical longevity of natural teeth,^31^ particularly among high-risk populations such as those with poor oral hygiene, sugary diets, and a genetic predisposition to oral diseases. Furthermore, the irreversible nature of dental erosion has dramatically challenged the clinical longevity of many natural tooth surfaces.

While the terms are sometimes used interchangeably, dental erosion refers to the chemical softening and irreversible loss of the outer surface of the teeth due to acid exposure,^28^ whereas erosive tooth wear is a broader term referring to the loss of tooth structure from the combined effects of chemical and mechanical processes.^38^ After an erosive challenge, the softened outer layer of mineral becomes susceptible to wear upon exposure to a sufficient level of physical interactions, such as attrition, together with abrasion. Dental abrasion is the physical loss of mineralised tooth substance caused by objects other than teeth,^38^ such as from a hard-bristle toothbrush.^40^

Compared to caries, the assessment of dental erosion has been difficult to model in controlled clinical trials. The irreversible nature of dental erosion has limited the number of studies conducted directly on the natural teeth of subjects.^18,48^ From an ethical standpoint, the permanent damage that can be caused by dental erosion led to the development of modified in situ erosion models that incorporated the use of removable appliances fitted with enamel specimens.^1,14,19,24,36,37^ The model simulates the process of dental erosion in an environment that includes natural plaque formation, saliva production, oral hygiene procedures, etc. The intent of the model is to mimic what occurs during the natural erosion process and provide clinically relevant information in a relatively short period of time. The removable appliances can be inserted in the mouth and worn for extended periods of time with controlled erosive acid challenges. Upon completion of these studies, specimens can be removed from the appliance and subjected to a variety of substrate analyses in the laboratory. Studies conducted using these approaches have successfully demonstrated the ability of various products to be partly resistant to chemically induced erosion.^13,20,36,42,45^ Among fluoride dentifrices, stannous fluoride has demonstrated significant anti-erosion benefits in these in situ models.^1,19,20,27,29,42,43,49^ Statistically significantly greater protection from erosion has been demonstrated for stannous fluoride-containing dentifrice vs various controls, include fluoride-free dentifrices and products containing sodium fluoride, sodium monofluorophosphate, and arginine.^1,19,20,25,36,37,42,43,48,49^

Over time, scientists and clinicians have incorporated increased levels of complexity into the various erosion models. While the initial versions of the models were often focused solely on erosion, later studies were modified in various ways to reflect the multifactorial nature of erosive tooth wear. For example, some research groups have incorporated abrasion into these models.^2,4,15,21,41^ Our group has recently expanded on our experience in the development and conducting of in situ erosion models^19,42,43^ to include an abrasive challenge, presented here. The goal is to better understand the effects of erosion plus abrasion, which we define here as an erosive tooth-wear model. Given stannous fluoride’s proven protection against erosion,^1,8-10,19, 20,25,27,29,32,36,37,42,43,47,48,49^ it was a natural candidate to evaluate in this new erosive tooth-wear model.

## Materials and Methods

### In Situ Method Description

**Fig 1:**
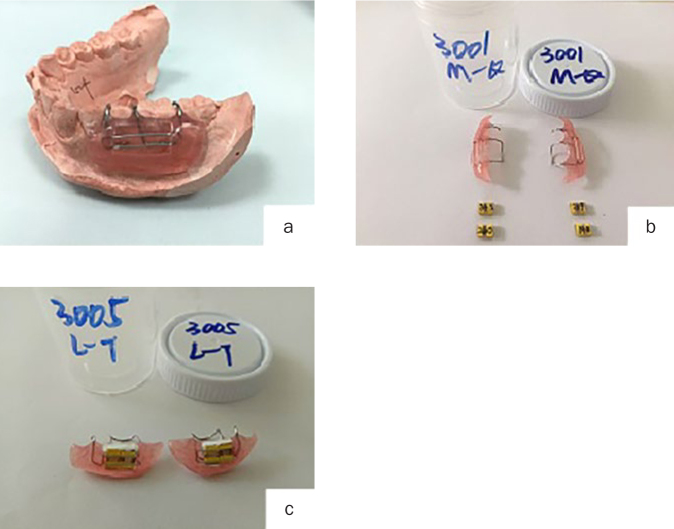
a. Mounting a mandibular appliance onto tooth surface; b. enamel specimens and appliance ready for combining; c. enamel specimens fitted into mandibular appliance, ready for insertion in the mouth.

The study, a variation of the previously published method of Hooper et al,^19^ was a randomised, controlled, parallel-group, clinical trial measuring erosion over 10 days. Mandibular buccal appliances containing 4 human enamel specimens were worn by 60 subjects (Fig 1). Ethical approval was granted by the Beijing Health Tech Research Co. Ltd. Institutional Review Board (study #CSD2016263). The study was designed and managed in compliance with the principles of good clinical practice.^30^

### Products Tested

Dentifrice products compared in this study included a conventional NaF dentifrice (Crest Cavity Protection dentifrice, The Procter & Gamble Company; Beijing, China) as the control dentifrice, containing 0.243% sodium fluoride (1100 ppm F), and an experimental, Sn-containing NaF dentifrice that contained 6100 ppm Sn (as stannous chloride) and 1450 ppm F (The Procter and Gamble Company). At screening, subjects were provided with a regular 0.243% sodium fluoride (1100 ppm F) marketed dentifrice (Crest Cavity Protection dentifrice) and manual toothbrush (Crest Wairouneigang manual toothbrush, The Procter & Gamble Company) for use at home until the follow-up visit. Subjects were required to use these products in place of their normal oral care products, twice per day (morning and evening) for the duration of the study. In addition, each participant was provided with an electric oscillating-rotating toothbrush (Oral-B Vitality Electric Toothbrush (D12), The Procter & Gamble Company) for use by clinic staff when power brushing was required.

### Subject Recruiting

Generally healthy subjects, 18 years of age or older, who met the inclusion criteria were recruited for the study. Key inclusion criteria consisted of a signed, informed consent document, the ability to understand and comply with directions, good general oral health and the ability to accommodate the mandibular intraoral in situ devices.

Potential subjects attended a screening visit before the start of the study. Prior to receiving any study-specific procedures, subjects were asked to read the participant information sheet and sign an informed consent. Demographic information and study entrance criteria were obtained, and an Oral Status Interview and Oral Examination were conducted for each subject. Subjects had an impression taken of their mandibular teeth and an appliance was constructed. Subjects who met all study entrance criteria were enrolled in the study and scheduled to return for a visit, during which all of the study procedures were explained in detail.

### Study Procedure

**Fig 2:**
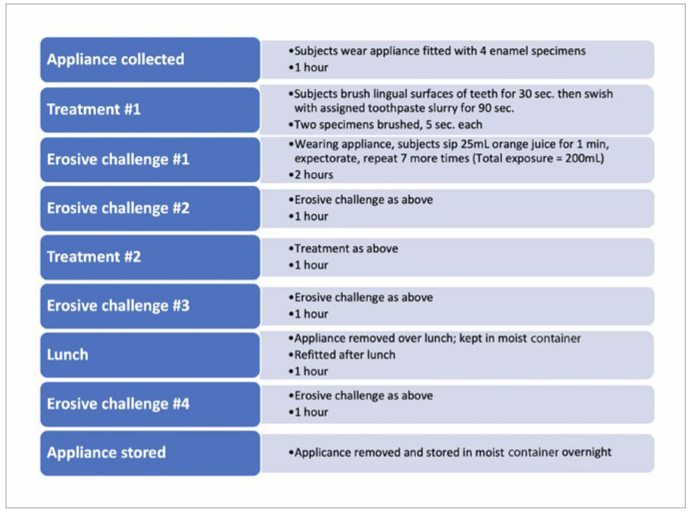
Flow of procedures followed on each of the 10 treatment days of the in situ study.

An outline of the daily procedure over the course of the 10 treatment days is included in Fig 2. Four prepared enamel samples (2 per side) were placed in the mandibular appliance of each subject (Fig 1). Treatment dentifrices were used twice per day and included 30 s of lingual brushing with a manual toothbrush followed by 90 s of oral slurry exposure. Specimens on the left (L) side of the mouth were then brushed for 5 s each by clinic staff with the electric oscillating-rotating toothbrush, while the specimens on the right side of the mouth were only exposed to the slurry treatment.

Four orange juice challenges using commercial orange juice (Uni-President Enterprises [China] Investment; Shanxi, China) were included each day. Subjects were asked to rinse their mouths (swish) with 25 ml of the orange juice (pH = 3.6) for 1 min, using a timer to keep track of time. After 1 min, subjects expectorated the juice. This procedure was repeated 7 additional times at each erosive challenge period, for a total exposure time of 8 min and a total volume of 200 ml of orange juice (8 x 25 ml increments). Enamel surfaces were measured using non-contact profilometry on day 10.

### Enamel Substrate Preparation and Measurement

Enamel samples were prepared from caries-free, human third molars that had been extracted and donated by patients aged 18 years and over before being cleaned, stored in a thymol solution and appropriately disinfected. The detailed preparation procedure is described in a previous publication.^49^

Post-treatment measurements were made using a non-contact profilometer (PS 50, Nanovea; Irvine, CA, USA) to measure step-height enamel loss.^33,49^ Prior to the start of the study, profilometry measurements were made on each enamel specimen to produce a flat surface with no more than an average profile of ±0.3 μm. Two pieces of PVC adhesive tape were placed parallel to each other to expose a window of enamel approximately 2 mm wide. The PVC adhesive tape covered areas served as baseline. PVC tapes were removed from the specimen after 10 days of treatment. The specimen was then placed onto the object stage of a non-contact profilometer (Nanovea P50) with an accuracy of 5.0 nm on the Z-axis, and the specimen surfaces were scanned at a sampling frequency of 300 Hz. Tissue loss was analysed and calculated using Professional 3D software (Digital Surf; Besançon, France).

### Statistical Efficacy Analyses

The study endpoint was loss of tooth surface, measured by profilometry on day 10. Since the day-10 enamel loss distribution is typically right-skewed in this model,^19,42,43,49^ average depths were analysed for treatment differences using a non-parametric method (Wilcoxon two-sample test). Statistical comparisons were two-sided at a 5% significance level.

### Safety

Oral safety evaluations included assessment of the oral soft and hard tissues. Assessment of the oral soft tissues was conducted by visual examination of the oral cavity and perioral area utilising a standard dental light, dental mirror and gauze. All abnormal findings noted after product assignment which were not documented at screening or were present at screening but had worsened during product usage, and which had the potential to be product-related, were recorded.

## Results

Sixty subjects were enrolled and all 60 completed the study with evaluable data. Of the 60 subjects, mean (SD) age was 41.98 years (10.27). The overall age range was 21-63 years.

At the day 10 visit, the adjusted mean (SE) enamel loss for specimens receiving slurry (erosion) treatment was 4.7 µm (0.61) (Sn-containing NaF) and 8.73 µm (1.12) (NaF control), with results demonstrating a statistically significant benefit of the Sn-containing dentifrice (46.2% benefit; p = 0.009). For specimens exposed to erosion/tooth-wear conditions, the enamel loss was 6.68 µm (1.29) (Sn-containing NaF) and 10.99 µm (1.29) (NaF group), which was statistically significant (p = 0.048; 39.2% better, in favour of the Sn-containing dentifrice). When data were combined, enamel loss (SE) for all specimens subjected to erosion + erosion/tooth wear was 5.61 µm (0.77) (Sn-containing NaF) and 9.9µm (1.3) (NaF group), with the difference again statistically significantly favouring the Sn-containing group (p = 0.022; 43.4% better) (Table 1).

**Table 1:** Results of the in situ clinical trial comparing the enamel tissue loss from NaF or Sn/NaF dentifrices after erosion (slurry) or erosive tooth wear (slurry + brushing) treatment protocols

Treatment protocol	Loss (µm)*		Sn/NaF vs NaF %	Sn/NaF vs NaF p-value
	NaF	Sn/NaF		
All	9.90 ± 1.30	5.61 ± 0.77	43.4	0.022
Slurry only	8.73 ± 1.12	4.70 ± 0.61	46.2	0.009
Slurry + brushing	10.99 ± 1.29	6.68 ± 1.29	39.2	0.048

*Adjusted mean enamel loss (mm) ± standard error.

Products included in the study were well tolerated. There were two (2) non-product related adverse events reported in the study.

## Discussion

As concerns about the widespread prevalence of dental erosion have grown in recent years, questions have been raised with respect to the relevance of models that are often used to assess erosion as well as the products intended to help alleviate its progression. Stannous fluoride dentifrice has been shown to protect against erosive challenges in various in situ models.^27,29^ An early model published by Hooper et al^19^ assessed erosion using twice daily toothpaste slurries and orange juice challenges 4x/day on human enamel samples over a 15-day period. Other studies have shown benefits for stannous fluoride dentifrice using shorter-term models, in as few as 5 days, and various acid challenges (e.g. grapefruit juice, orange juice, citric acid).^25,27^

While standard erosion models focus entirely on the potential for various technologies or products to protect enamel against erosive acid damage, erosive tooth-wear models are intended to take into account an additional factor in the overall tooth-loss equation when the effects of abrasive activity are also considered. Studies have demonstrated that significantly greater tooth-surface loss can occur when an abrasive factor, in addition to erosion, is included in a study without efficacious protective treatments. In a 15-day in situ study, Seong et al^39^ demonstrated that the inclusion of abrasion significantly increased tissue loss compared to erosion alone in the absence of any protective treatments. Other erosion + abrasion studies^4,6,11^ included assessments of different protective treatments and found that, with these treatments, tissue loss was greater when abrasive forces were present. While these studies focused on erosion + abrasion, they were limited to measuring effects of NaF or NaF plus calcium-based abrasives.

A few studies have included erosion and abrasion as well as an evaluation of Sn-containing products. Like their erosion-only counterparts in which stannous formulations have been demonstrated to be highly effective, these erosion/tooth-wear studies have also demonstrated protective benefits for the stannous-based formulations, whether the tested products were in the form of a rinse,^7,46^ gel,^23^ or toothpaste,^5,22,36,37^ although not every stannous formulation tested provided the same level of benefit in each of the different studies. Study conditions and products varied significantly between the various models, with some incorporating brushing of specimens for 5 s at a force of 2.5 N,^36,37^ use of an electric toothbrush 2x/day for 15 s per treatment^5^ or brushing of specimens for 30 s at each treatment.^22^ Erosive challenges also differed significantly: Schlueter et al^36,37^ included 6 daily challenges using 0.5% citric acid, pH 2.6, for 2 min per day, Comar et al^5^ included an erosive challenge with a commercial product, Sprite Zero (Coca-Cola China; Shanghai, China), for 4 x 90 s per day, and Hove et al^22^ directly etched specimens for 2 min, twice per day, with 0.01M HCl, a condition intended to mimic gastric reflux/vomiting. Importantly, regardless of how these studies were conducted, results overwhelmingly favoured the ability of stannous-containing products to provide significantly greater erosive tooth-wear protection compared to conventional fluoride products containing only NaF.

The treatment cycle incorporated into the current study included two series of product treatments followed by two acid exposures, which simulated exposure to dietary acid challenges and twice-daily oral hygiene over the course of each treatment day (Fig 2). The design of this study included direct brushing of specimens on one side of the mouth, with treatment on the other side limited to the erosive factor. This allowed a comparison between erosive and erosive tooth wear conditions. To imitate tooth-wear conditions, specimens were brushed for a period of 5 s each.^46^

One potential limitation of the research is that the Sn-containing dentifrice was formulated with 1450 ppm F, while the control dentifrice contained 1100 ppm F as NaF. However, while this slightly higher level of fluoride may provide positive effects in caries models, NaF is not generally associated with significant erosion protection, whereas dentifrices containing either SnF_2_ or SnCl_2_ have been recognised for their ability to help prevent dental erosion.^3^ This is noted in the Consensus report of the European Federation of Conservative Dentistry, erosive tooth wear-diagnosis and management: ‘Products (e.g. toothpastes or mouth rinses) containing stannous fluoride or stannous chloride have the potential for slowing the progression of ETW. For other products, data so far are sparse.’^3^ Results from this study are consistent with previous studies, both in vitro,^10^ in which a Sn-containing NaF dentifrice formulated with 1450 ppm F performed significantly better than both 1100 ppm F (NaF) and 1450 ppm F (NaF) dentifrices, which were not significantly different from each other, and in situ,^20^ where a Sn-containing NaF dentifrice (1450 ppm F) performed significantly better than a product formulated with 1450 ppm F (NaF). Moreover, in vitro studies have shown that products containing up to 5000 ppm F as NaF still do not perform as well as 0.454% stannous fluoride dentifrice.^8^

During brushing, NaF and SnCl_2_ combine in a synergistic fashion, forming a bioavailable, stannous-fluoride complex,^17^ with the end result being the precipitation of an invisible barrier layer comprised of either stannous fluorophosphate or stannous oxide on the surface of treated teeth. This deposited barrier layer then serves as the mechanism for protection against erosive acid challenges. In this study, regardless of whether erosion or erosion/tooth wear were considered, the statistical conclusions remained consistent and in agreement with previous studies that demonstrated the superior performance of stannous-containing formulations. New stannous-containing dentifrices have been recently introduced on the market by different manufacturers.^16^ Since composition is critically important to achieve the bioavailability of the active ingredient, future research could evaluate these formulations for erosive tooth-wear protection.

## Conclusion

In this research, the Sn-containing dentifrice demonstrated significantly better erosion protection under both slurry (erosion) and slurry + brushing (erosive tooth wear) conditions (p < 0.05), compared to the conventional NaF dentifrice. Of primary importance in reducing erosive tooth wear is reduction of acid damage. This research demonstrates that with properly formulated stannous-containing dentifrices, clinical methods developed based on acid erosion alone are likely adequate to predict both erosion-only and erosive/tooth wear results and reaffirms the importance of using a Sn-containing dentifrice to help prevent tooth wear from dietary acid exposure.
